# Analysis of MicroRNA‐502‐3p and MicroRNA‐501‐3p in the Cerebrospinal Fluid of Alzheimer’s Disease and its Corresponding Neuropathology: Potential Biomarkers

**DOI:** 10.1002/alz.087657

**Published:** 2025-01-09

**Authors:** Davin Devara, Bhupender Sharma, Subodh Kumar, Melissa Torres, Sheryl Rodriguez, Daniela Rodarte

**Affiliations:** ^1^ Texas Tech University Health Sciences Center El Paso, El Paso, TX USA

## Abstract

**Background:**

Alzheimer’s disease (AD) continues to be the leading cause of dementia. Few treatment options exist to manage AD. It is essential to diagnose AD early to slow its progression; however, there are very limited diagnostic tools to accurately diagnose AD. MicroRNAs (MiRNAs) are currently being studied for their biomarker potential in many diseases, including AD. This study explores the biomarker potential of miR‐502‐3p and miR‐501‐3p in the cerebrospinal fluid (CSF) exosomes of AD patients and their relationship to the severity of amyloid plaques and neurofibrillary tangles (NFTs) in affected brain regions.

**Method:**

CSF samples from AD patients (n=26) and unaffected controls (UC) (n=10) were obtained from NIH NeuroBioBank. We then isolated exosomes from CSF and analyzed the expression of miR‐502‐3p and miR‐501‐3p. Results were compared with CSF Aβ1‐40, Aβ1‐42, Tau and p‐Tau levels. We also evaluated the relationship of miR‐502‐3p and miR‐501‐3p with the neuropathology reports of the CSF samples.

**Result:**

MiR‐502‐3p and miR‐501‐3p expression levels were upregulated in the AD CSF exosomes relative to the UC CSF exosomes. MiR‐502‐3p level was positively correlated with CSF Aβ1‐40 level while miR‐501‐3p was positively correlated with CSF p‐Tau levels. The expressions of miR‐502‐3p and miR‐501‐3p were significantly associated with the severity of amyloid plaques and NFTs in the entorhinal cortex, hippocampus, amygdala, middle frontal gyrus, inferior parietal lobule, and superior temporal gyrus.

**Conclusion:**

MiR‐502‐3p and miR‐501‐3p could potentially be useful CSF biomarkers for AD in the future. They may also be used to track the progression of AD in patients.

